# Scale dependency in native–exotic richness relationships revisited

**DOI:** 10.1002/ece3.8549

**Published:** 2022-01-12

**Authors:** Qinfeng Guo

**Affiliations:** ^1^ USDA FS Eastern Forest Environmental Threat Assessment Center Research Triangle Park North Carolina USA

**Keywords:** biotic resistance, diversity, invasibility, model, species invasion

## Abstract

In their seminal paper, Shea and Chesson (Trends in Ecology & Evolution, 2002, 17, 170) developed a highly cited model (S&C model) showing scale dependency in the native–exotic richness relationships. Two decades later, extensive additional data have been accumulated, leading to new findings and insights. Accordingly, two updates were made here to the original S&C model: (1) changing the “negative” richness relationship between natives and exotics to “non‐consistent” or “non‐significant”; and (2) modifying the original diagram to correctly represent native and exotic species richness and their correlations across both small and large scales.

## Graphical abstract

I revised the original S&C model based on two updates: (1) changing the “negative” richness relationship between natives and exotics to “no consistent” or “non‐significant”; and (2) modifying the original diagram that could correctly represent native and exotic species richness and their correlations across both small and large scales.

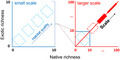

Species diversity has long been perceived as a major driving force in invasion resistance (Elton, 1958; Lonsdale, 1999; Williamson, 1996). Two decades ago, in their effort to reconcile the “relationships between invasion success and species richness on different spatial scales,” Shea and Chesson (Shea & Chesson, 2002) (S&C thereafter) developed a model showing scale dependency in native–exotic richness relationships. Ever since its publication, many theoretical and empirical studies have cited their model and made a similar statement that the native–exotic richness relationships are negative on small scales but positive on large scales (Davies et al., 2005; Fridley et al., 2004).

In the past two decades, much progress has been made in biotic invasion research, especially relating to scale dependency. The new studies often use more complete and extensively accumulated data. As researchers continue to assess habitat invasibility around the globe using the same or similar models based on recent developments and findings, it is time to update this important model with new findings and insights. Particularly, an increasing number of studies (Gilbert & Lechowicz, 2005; Hill & Fischer, 2014; Sandel & Corbin, 2010) found no or even positive native–exotic richness correlations on small scales. A recent synthesis (Guo, 2015) thus found no consistent native–exotic richness relationship across small scales (see also Burns, 2016; Tomasetto et al., 2019; Valone & Weyers, 2019). For these reasons, here I have modified the original S&C model by providing two main updates.

First, recent studies show that there is no consistent or significant negative native–exotic relationship on small scales, especially in real‐world settings (Guo, 2015; Jeschke et al., 2012; Von Holle, 2013). Because of this, and because the S&C model will continue to be cited frequently, it is now necessary to correct the “negative native–exotic richness relationship” misperception.

Second, the original S&C model is somewhat imprecise as it used the same set of axes to represent native and exotic richness for both small and large scales (the assumed data points representing the two scales with very different richness values overlapped in the diagram). Following the well‐established species–area relationship (MacArthur & Wilson, 1967), the richness values would be much higher over large scales than over small scales; thus, the same *x*‐ and *y*‐axes cannot represent species richness across both small and large scales. In other words, in their diagram, the richness data at small scales showing negative native–exotic relationships cannot be placed at the upper‐right corner as the small‐scale species richness would be lower. Data points representing small scales cannot have very high species richness unless the different groups of data points represent species‐poor (in the bottom‐left corner) and species‐rich (in the upper‐right corner) habitats.

For the two reasons above, I modified the S&C model by changing the “negative” richness relationship between natives and exotics to “non‐significant” (Figure 1 left). As the scale increases, the relationships become increasingly positive to reflect the patterns frequently observed in both theoretical and observational studies (Figure 1 right).

**FIGURE 1:**
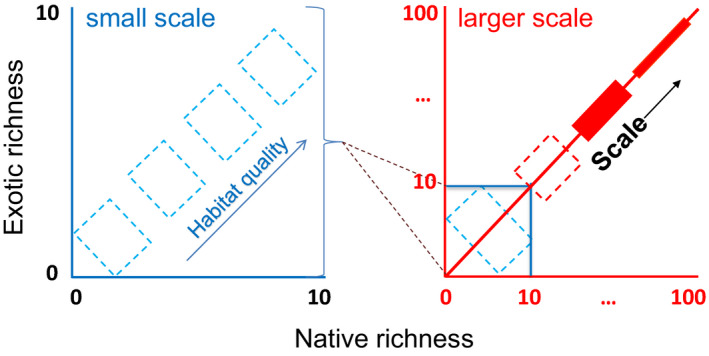
A comparison of the native–exotic richness relationships or correlation across small vs. large scales. The revised S&C model separates between small (blue) and large scales (red) because the species richness levels between the two sets of scales would be very different across similar habitats unless very different habitats (e.g., polar habitats or deserts vs. tropical rain forests) are included. Dashed boxes indicate insignificant correlations when richness data are used. Left: on small scales, the native–exotic richness relationships are likely negative but often not significant. However, the native–exotic relationships are likely to be significantly negative if plant biomass, cover, or density measures are used (see Figure 3). Right: on large scales, the native–exotic richness correlations (not necessarily relationship) are mostly positive

Alternatively, for visualization purpose, we can add a second set of axes to the original S&C model that can show patterns over large scales (Figure 2). This method is useful if we compare the same type of habitats because the high species richness in the upper‐right corner over large scale cannot be embedded with small‐scale patterns in which the species richness values should be much lower.

**FIGURE 2:**
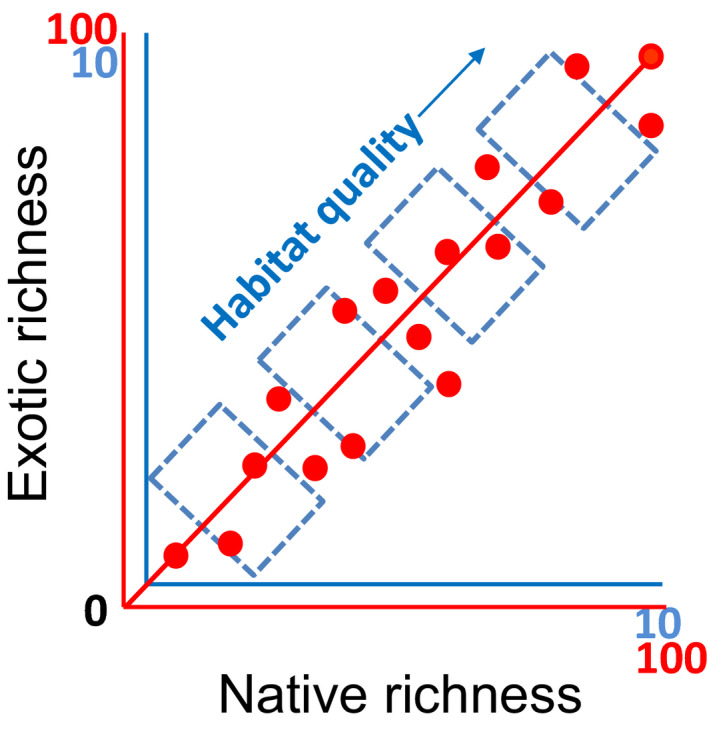
A comparison of the native–exotic richness relationships or correlation across small vs. large scales. Two sets of axes are used to represent species richness on small (0–10 species—blue) vs. large (0–100 species—red) scales

In the past, most studies have used the number of exotic species as an indicator of habitat invasibility, especially at large scales (Fridley et al., 2004; Herben et al., 2004; Lonsdale, 1999). On small scales, most studies showing the biotic resistance to invasions owing to native diversity actually used biomass, survivorship, size, or density of exotics (either a single invader or all exotics) rather than “richness” as the dependent variable (e.g., Figure 3) (Levine & D'Antonio, 1999). Also, many of the theoretical studies that report negative native–exotic relationships are based on Lotka‐Volterra models (i.e., the response variable is not exotic richness).

**FIGURE 3:**
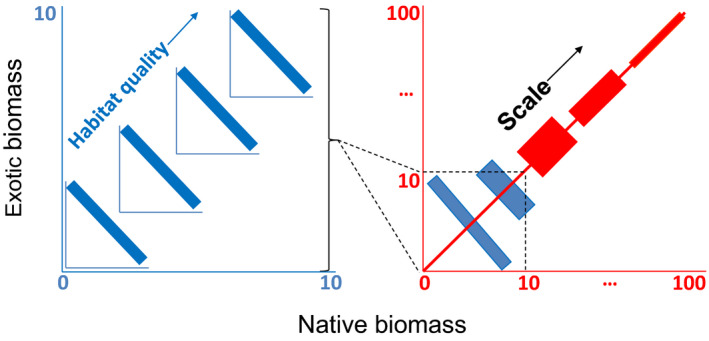
An example of native–exotic relationships or correlation across small (mostly negative; left) vs. large scales (positive; right). Here, plant abundance (biomass, cover, density) rather than richness is used (see also Guo & Symstad, 2008)

In short, while the positive correlations between native and exotic richness over large scales are indeed common and have been attributed to external factors such as area (space) and resources that similarly control both native and exotic species (Fridley et al., 2007) or null models (Fridley et al., 2004), it is time to eliminate the frequently claimed “negative small‐scale native–exotic richness relationships” perception. On small scales, native richness could indeed enhance invasion resistance by reducing the invaders' performance (abundance and distribution), but it rarely reaches the level required to expel exotic species. This is especially the case when the habitats are disturbed. The negative native–exotic richness relationship observed in a few experiments might be caused directly by higher biomass or density in species‐richer habitats—at least right after initial planting/seeding rather than by higher species richness itself (Herben et al., 2004; Smith & Cote, 2019).

## CONFLICT OF INTEREST

The author declares no conflict of interest.

## AUTHOR CONTRIBUTIONS

**Qinfeng Guo**: Conceptualization (equal); investigation (equal); methodology (equal); project administration (equal); visualization (equal); writing—original draft (equal); writing—review and editing (equal).

## Data Availability

Data sharing not applicable—no new data generated.

## References

[ece38549-bib-0001] Burns, K. (2016). Native–exotic richness relationships: A biogeographic approach using turnover in island plant populations. Ecology, 97, 2932–2938. 10.1002/ecy.1579 27870029

[ece38549-bib-0002] Davies, K. F. , Chesson, P. , Harrison, S. , Inouye, B. D. , Melbourne, B. A. , & Rice, K. J. (2005). Spatial heterogeneity explains the scale dependence of the native‐exotic diversity relationship. Ecology, 86, 1602–1610. 10.1890/04-1196

[ece38549-bib-0003] Elton, C. S. (1958). The ecology of invasions by plants and animals. Methuen.

[ece38549-bib-0004] Fridley, J. D. , Brown, R. L. , & Bruno, J. F. (2004). Null models of exotic invasion and scale‐dependent patterns of native and exotic species richness. Ecology, 85, 3215–3222. 10.1890/03-0676

[ece38549-bib-0005] Fridley, J. D. , Stachowicz, J. J. , Naeem, S. , Sax, D. , Seabloom, E. , Smith, M. , Stohlgren, T. , Tilman, D. , & Holle, B. V. (2007). The invasion paradox: Reconciling pattern and process in species invasions. Ecology, 88, 3–17. 17489447 10.1890/0012-9658(2007)88[3:tiprpa]2.0.co;2

[ece38549-bib-0006] Gilbert, B. , & Lechowicz, M. J. (2005). Invasibility and abiotic gradients: The positive correlation between native and exotic plant diversity. Ecology, 86, 1848–1855. 10.1890/04-09997

[ece38549-bib-0007] Guo, Q. (2015). No consistent small‐scale native‐exotic relationships. Plant Ecology, 216, 1225–1230. 10.1007/s11258-015-0503-7

[ece38549-bib-0008] Guo, Q. , & Symstad, A. (2008). A two‐part measure of degree of invasion for cross‐community comparisons. Conservation Biology, 22, 666–672. 10.1111/j.1523-1739.2008.00915.x 18477032

[ece38549-bib-0009] Herben, T. , Mandák, B. , Bímová, K. , & Münzbergová, Z. (2004). Invasibility and species richness of a community: A neutral model and a survey of published data. Ecology, 85, 3223–3233. 10.1890/03-0648

[ece38549-bib-0010] Hill, K. C. , & Fischer, D. G. (2014). Native–exotic species richness relationships across spatial scales in a prairie restoration matrix. Restoration Ecology, 22, 204–213. 10.1111/rec.12067

[ece38549-bib-0011] Jeschke, J. M. , Gómez Aparicio, L. , Haider, S. , Heger, T. , Lortie, C. J. , Pyšek, P. , & Strayer, D. L. (2012). Support for major hypotheses in invasion biology is uneven and declining. NeoBiota, 14, 1–20. 10.3897/neobiota.14.3435

[ece38549-bib-0012] Levine, J. M. , & D'Antonio, C. M. (1999). Elton revisited: A review of evidence linking diversity and invasibility. Oikos, 87(1), 15–26. 10.2307/3546992

[ece38549-bib-0013] Lonsdale, W. M. (1999). Global patterns of plant invasions and the concept of invasibility. Ecology, 80, 1522–1536.

[ece38549-bib-0014] MacArthur, R. H. , & Wilson, E. O. (1967). The theory of island biogeography. Princeton University Press.

[ece38549-bib-0015] Sandel, B. , & Corbin, J. D. (2010). Scale, disturbance and productivity control the native‐exotic richness relationship. Oikos, 119, 1281–1290. 10.1111/j.1600-0706.2010.18230.x

[ece38549-bib-0016] Shea, K. , & Chesson, P. (2002). Community ecology theory as a framework for biological invasions. Trends in Ecology & Evolution, 17, 170–176. 10.1016/S0169-5347(02)02495-3

[ece38549-bib-0017] Smith, N. S. , & Cote, I. M. (2019). Multiple drivers of contrasting diversity–invasibility relationships at fine spatial grains. Ecology, 100, e02573. 10.1002/ecy.2573 30516274

[ece38549-bib-0018] Tomasetto, F. , Duncan, R. P. , & Hulme, P. E. (2019). Resolving the invasion paradox: Pervasive scale and study dependence in the native‐alien species richness relationship. Ecology Letters, 22, 1038–1046. 10.1111/ele.13261 30920165

[ece38549-bib-0019] Valone, T. J. , & Weyers, D. P. (2019). Invasion intensity influences scale‐dependent effects of an exotic species on native plant diversity. Scientific Reports, 9, 1–8. 10.1038/s41598-019-55165-z 31822718 PMC6904574

[ece38549-bib-0020] Von Holle, B. (2013). Environmental stress alters native‐nonnative relationships at the community scale. Biological Invasions, 15, 417–427. 10.1007/s10530-012-0297-7

[ece38549-bib-0021] Williamson, M. (1996). Biological invasions. Chapman and Hall.

